# 2-Bromo-*N*-(4-chloro­phen­yl)-2-methyl­propanamide

**DOI:** 10.1107/S1600536811035562

**Published:** 2011-09-14

**Authors:** Rodolfo Moreno-Fuquen, David E. Quintero, Fabio Zuluaga, Alan R. Kennedy, Regina H. De Almeida Santos

**Affiliations:** a Departamento de Química – Facultad de Ciencias, Universidad del Valle, Apartado 25360, Santiago de Cali, Colombia; b WestCHEM, Department of Pure and Applied Chemistry, University of Strathclyde, 295 Cathedral Street, Glasgow G1 1XL, Scotland; c Instituto de Química de São Carlos, Universidade de São Paulo, USP, São Carlos, SP, Brazil

## Abstract

In the title mol­ecule, C_10_H_11_BrClNO, there is a twist between the mean plane of the amide group and the benzene ring [C(=O)—N—C—C torsion angle = −27.1 (3)°]. In the crystal, inter­molecular N—H⋯O and weak C—H⋯O hydrogen bonds link the  mol­ecules into chains along [010].

## Related literature

For initiators in ATRP processes (polymerization by atom transfer radical), see: Matyjaszewski & Xia (2001 ▶); Pietrasik & Tsarevsky (2010 ▶). For end-functionalized linear polymers, see: Matyjaszewski & Mueller (2008 ▶); Stenzel-Rosenbaum *et al.* 2001 ▶). For hydrogen-bond graph-set motifs, see: Etter (1990 ▶). For hydrogen bonding, see: Nardelli (1995 ▶).
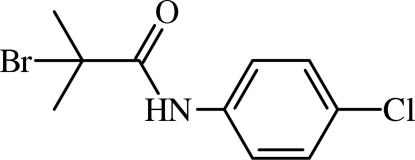


## Experimental

### 

#### Crystal data

C_10_H_11_BrClNO
*M* *_r_* = 276.56
Orthorhombic, 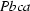

*a* = 9.7449 (3) Å
*b* = 10.1063 (3) Å
*c* = 22.8803 (7) Å
*V* = 2253.36 (12) Å^3^
*Z* = 8
Mo *K*α radiation
μ = 3.85 mm^−1^
*T* = 123 K
0.45 × 0.22 × 0.08 mm

#### Data collection

Oxford Diffraction Gemini S diffractometer
Absorption correction: multi-scan (*CrysAlis PRO*; Oxford Diffraction, 2009 ▶) *T*_min_ = 0.387, *T*_max_ = 1.000
9676 measured reflections
2684 independent reflections
2225 reflections with *I* > 2σ(*I*)
*R*_int_ = 0.028
Standard reflections: 0

#### Refinement

*R*[*F* ^2^ > 2σ(*F* ^2^)] = 0.032
*wR*(*F* ^2^) = 0.069
*S* = 1.05
2684 reflections
133 parameters
H atoms treated by a mixture of independent and constrained refinement
Δρ_max_ = 1.00 e Å^−3^
Δρ_min_ = −0.60 e Å^−3^

### 

Data collection: *CrysAlis CCD* (Oxford Diffraction, 2009 ▶); cell refinement: *CrysAlis CCD*; data reduction: *CrysAlis RED* (Oxford Diffraction, 2009 ▶); program(s) used to solve structure: *SHELXS97* (Sheldrick, 2008 ▶); program(s) used to refine structure: *SHELXL97* (Sheldrick, 2008 ▶); molecular graphics: *ORTEP-3 for Windows* (Farrugia, 1997 ▶) and *Mercury* (Macrae *et al.*, 2006 ▶); software used to prepare material for publication: *WinGX* (Farrugia, 1999 ▶).

## Supplementary Material

Crystal structure: contains datablock(s) I, global. DOI: 10.1107/S1600536811035562/hg5088sup1.cif

Structure factors: contains datablock(s) I. DOI: 10.1107/S1600536811035562/hg5088Isup2.hkl

Supplementary material file. DOI: 10.1107/S1600536811035562/hg5088Isup3.cml

Additional supplementary materials:  crystallographic information; 3D view; checkCIF report

## Figures and Tables

**Table 1:** Hydrogen-bond geometry (Å, °)

*D*—H⋯*A*	*D*—H	H⋯*A*	*D*⋯*A*	*D*—H⋯*A*
N1—H1*N*⋯O1^i^	0.81 (3)	2.17 (3)	2.972 (2)	169 (3)
C10—H10⋯O1^i^	0.95	2.71	3.433 (3)	133
C4—H4*B*⋯O1^i^	0.98	2.53	3.453 (3)	158

Symmetry code: (i) 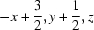.

**Fig. 1.**
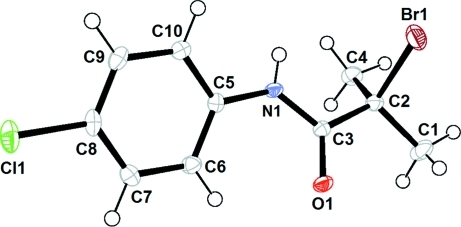
An ORTEP-3 (Farrugia, 1997) plot of (I) with displacement ellipsoids drawn at the 50% probability level. H atoms are shown as spheres of arbitrary radius.

**Fig. 2.**
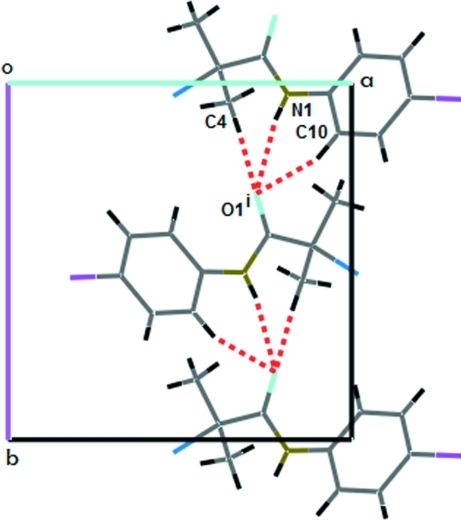
Part of the crystal structure of (I), showing the formation of a one dimensional chain along [010]. Symmetry code: (i) -x + 3/2,+y + 1/2,+z

## supplementary crystallographic information

### Comment

The title compound (I), is a monofunctional alkyl halyde derivative, which can be used as an initiator in Atom Transfer Radical Polymerization processes (ATRP) (Matyjaszewski & Xia, 2001; Pietrasik & Tsarevsky, 2010). This derivative can form end-functionalized linear polymers when used as an initiator (Matyjaszewski *et al.* 2008; Stenzel-Rosenbaum *et al.* 2001). The molecular structure of (I) is shown in Fig. 1. There is a twist between the mean plane of the amide group and benzene ring giving a C3—N1—C5—C6 torsion angle of -27.1 (3)°. The crystal structure is stabilized by intermolecular N—H···O and weak C—H···O hydrogen bonds (see Table 1, Nardelli, 1995). Indeed, molecules of (I) are linked by N1—H1N···O1^i^, C10—H10···O1^i^ and C4—H4B···O1^i^ hydrogen bonds (i: -*x* + 3/2,+*y* + 1/2,+*z*) which lead to the formation of C(4) (Etter, 1990) one dimensional chain along [010] (Fig. 2).

### Experimental

The initial reagents were purchased from Aldrich Chemical Co. and were used as received. In a 100 mL round bottom flask 4-chloroaniline (2.315 mmoles, 0.295 g), triethylamine (0.463 mmol, 0.027 g) were mixed, then a solution of 2-bromo isobutyryl bromide (0.450 g) in anhydrous THF (5 ml) was added drop wise, under an argon stream. The reaction was carried out in a dry bag overnight under magnetic stirring. The solid was filtered off and dichloromethane (20 ml) added to the organic phase which was washed with brine (50 ml) followed by water (10 ml). The solution was concentrated at low pressure affording colourless crystals and recrystalized from a solution of hexane and ethyl acetate (80:20). *M*.p. 386 (1) K.

### Refinement

The H-atoms were positioned geometrically [C—H= 0.95 Å for aromatic and C—H= 0.98 Å for methyl, and with *U*_iso_(H) (1.2 and 1.5 times *U*_eq_ of the parent atom respectivelly]. The amide-H1N atom was located in a difference Fourier map and was refined freely.

### Crystal data

**Table:**

C_10_H_11_BrClNO	*D*_x_ = 1.630 Mg m^−^^3^
*M**_r_* = 276.56	Melting point: 386(1) K
Orthorhombic, *P**b**c**a*	Mo *K*α radiation, λ = 0.71073 Å
Hall symbol: -P 2ac 2ab	Cell parameters from 4107 reflections
*a* = 9.7449 (3) Å	θ = 2.9–29.7°
*b* = 10.1063 (3) Å	µ = 3.85 mm^−^^1^
*c* = 22.8803 (7) Å	*T* = 123 K
*V* = 2253.36 (12) Å^3^	Bar, colourless
*Z* = 8	0.45 × 0.22 × 0.08 mm
*F*(000) = 1104	

### Data collection

**Table:**

Oxford Diffraction Gemini S diffractometer	2684 independent reflections
Radiation source: fine-focus sealed tube	2225 reflections with *I* > 2σ(*I*)
graphite	*R*_int_ = 0.028
ω scans	θ_max_ = 28.0°, θ_min_ = 3.0°
Absorption correction: multi-scan (*CrysAlis PRO*; Oxford Diffraction, 2009)	*h* = −12→12
*T*_min_ = 0.387, *T*_max_ = 1.000	*k* = −11→13
9676 measured reflections	*l* = −29→30

### Refinement

**Table:**

Refinement on *F*^2^	Primary atom site location: structure-invariant direct methods
Least-squares matrix: full	Secondary atom site location: difference Fourier map
*R*[*F*^2^ > 2σ(*F*^2^)] = 0.032	Hydrogen site location: inferred from neighbouring sites
*wR*(*F*^2^) = 0.069	H atoms treated by a mixture of independent and constrained refinement
*S* = 1.05	*w* = 1/[σ^2^(*F*_o_^2^) + (0.0275*P*)^2^ + 1.8897*P*] where *P* = (*F*_o_^2^ + 2*F*_c_^2^)/3
2684 reflections	(Δ/σ)_max_ < 0.001
133 parameters	Δρ_max_ = 1.00 e Å^−^^3^
0 restraints	Δρ_min_ = −0.60 e Å^−^^3^

### Special details

**Table:**

Geometry. All e.s.d.'s (except the e.s.d. in the dihedral angle between two l.s. planes) are estimated using the full covariance matrix. The cell e.s.d.'s are taken into account individually in the estimation of e.s.d.'s in distances, angles and torsion angles; correlations between e.s.d.'s in cell parameters are only used when they are defined by crystal symmetry. An approximate (isotropic) treatment of cell e.s.d.'s is used for estimating e.s.d.'s involving l.s. planes.
Refinement. Refinement of *F*^2^ against ALL reflections. The weighted *R*-factor *wR* and goodness of fit *S* are based on *F*^2^, conventional *R*-factors *R* are based on *F*, with *F* set to zero for negative *F*^2^. The threshold expression of *F*^2^ > σ(*F*^2^) is used only for calculating *R*-factors(gt) *etc*. and is not relevant to the choice of reflections for refinement. *R*-factors based on *F*^2^ are statistically about twice as large as those based on *F*, and *R*- factors based on ALL data will be even larger.

### Fractional atomic coordinates and isotropic or equivalent isotropic displacement parameters (Å^2^)

**Table:**

	*x*	*y*	*z*	*U*_iso_*/*U*_eq_	
Br1	0.48117 (2)	0.03662 (3)	0.143191 (11)	0.02744 (9)	
Cl1	1.31938 (6)	0.04546 (7)	−0.00111 (2)	0.02798 (15)	
O1	0.77815 (16)	−0.19000 (14)	0.15413 (7)	0.0189 (3)	
N1	0.81397 (19)	0.03082 (19)	0.14313 (8)	0.0150 (4)	
C1	0.5483 (3)	−0.1697 (2)	0.22170 (11)	0.0275 (6)	
H1A	0.6122	−0.2144	0.2483	0.041*	
H1B	0.5265	−0.2286	0.1889	0.041*	
H1C	0.4639	−0.1476	0.2428	0.041*	
C2	0.6140 (2)	−0.0437 (2)	0.19856 (9)	0.0168 (5)	
C3	0.7429 (2)	−0.0750 (2)	0.16220 (9)	0.0138 (4)	
C4	0.6432 (3)	0.0534 (2)	0.24801 (10)	0.0215 (5)	
H4A	0.5595	0.0670	0.2710	0.032*	
H4B	0.6734	0.1381	0.2316	0.032*	
H4C	0.7154	0.0175	0.2733	0.032*	
C5	0.9350 (2)	0.0288 (2)	0.10845 (9)	0.0139 (4)	
C6	1.0272 (2)	−0.0771 (2)	0.10905 (10)	0.0176 (5)	
H6	1.0088	−0.1532	0.1321	0.021*	
C7	1.1457 (2)	−0.0702 (2)	0.07567 (10)	0.0193 (5)	
H7	1.2092	−0.1415	0.0761	0.023*	
C8	1.1717 (2)	0.0402 (2)	0.04186 (9)	0.0191 (5)	
C9	1.0812 (2)	0.1457 (2)	0.04075 (9)	0.0196 (5)	
H9	1.0999	0.2211	0.0173	0.024*	
C10	0.9626 (2)	0.1399 (2)	0.07439 (10)	0.0172 (5)	
H10	0.9000	0.2120	0.0742	0.021*	
H1N	0.778 (3)	0.103 (3)	0.1467 (11)	0.024 (7)*	

### Atomic displacement parameters (Å^2^)

**Table:**

	*U*^11^	*U*^22^	*U*^33^	*U*^12^	*U*^13^	*U*^23^
Br1	0.01641 (13)	0.03536 (16)	0.03055 (14)	0.00118 (10)	−0.00217 (9)	0.00496 (12)
Cl1	0.0173 (3)	0.0437 (4)	0.0230 (3)	−0.0036 (3)	0.0062 (2)	0.0048 (3)
O1	0.0206 (8)	0.0105 (7)	0.0256 (8)	−0.0002 (6)	0.0060 (6)	−0.0013 (6)
N1	0.0178 (9)	0.0096 (9)	0.0176 (9)	0.0016 (8)	0.0048 (7)	−0.0001 (8)
C1	0.0317 (14)	0.0173 (12)	0.0335 (14)	−0.0036 (10)	0.0161 (11)	0.0016 (11)
C2	0.0172 (11)	0.0139 (11)	0.0194 (10)	0.0012 (9)	0.0025 (9)	0.0004 (9)
C3	0.0155 (10)	0.0133 (10)	0.0125 (9)	−0.0004 (8)	−0.0016 (8)	0.0008 (8)
C4	0.0259 (12)	0.0177 (11)	0.0210 (11)	0.0022 (10)	0.0066 (9)	−0.0044 (10)
C5	0.0141 (10)	0.0158 (10)	0.0117 (9)	−0.0024 (9)	−0.0006 (8)	−0.0027 (9)
C6	0.0203 (11)	0.0152 (10)	0.0171 (10)	0.0004 (9)	0.0016 (9)	0.0022 (9)
C7	0.0166 (11)	0.0214 (12)	0.0199 (11)	0.0026 (9)	0.0017 (9)	−0.0019 (9)
C8	0.0135 (10)	0.0295 (13)	0.0142 (10)	−0.0053 (10)	0.0017 (8)	−0.0013 (10)
C9	0.0217 (12)	0.0217 (12)	0.0155 (10)	−0.0072 (10)	−0.0008 (9)	0.0036 (9)
C10	0.0186 (11)	0.0153 (11)	0.0178 (10)	−0.0005 (9)	−0.0002 (9)	0.0008 (9)

### Geometric parameters (Å, °)

**Table:**

Br1—C2	1.985 (2)	C4—H4B	0.9800
Cl1—C8	1.744 (2)	C4—H4C	0.9800
O1—C3	1.226 (2)	C5—C10	1.393 (3)
N1—C3	1.346 (3)	C5—C6	1.397 (3)
N1—C5	1.422 (3)	C6—C7	1.387 (3)
N1—H1N	0.81 (3)	C6—H6	0.9500
C1—C2	1.521 (3)	C7—C8	1.381 (3)
C1—H1A	0.9800	C7—H7	0.9500
C1—H1B	0.9800	C8—C9	1.384 (3)
C1—H1C	0.9800	C9—C10	1.390 (3)
C2—C4	1.524 (3)	C9—H9	0.9500
C2—C3	1.539 (3)	C10—H10	0.9500
C4—H4A	0.9800		
			
C3—N1—C5	126.59 (19)	C2—C4—H4C	109.5
C3—N1—H1N	117.2 (19)	H4A—C4—H4C	109.5
C5—N1—H1N	115.3 (19)	H4B—C4—H4C	109.5
C2—C1—H1A	109.5	C10—C5—C6	119.9 (2)
C2—C1—H1B	109.5	C10—C5—N1	117.43 (19)
H1A—C1—H1B	109.5	C6—C5—N1	122.59 (19)
C2—C1—H1C	109.5	C7—C6—C5	119.4 (2)
H1A—C1—H1C	109.5	C7—C6—H6	120.3
H1B—C1—H1C	109.5	C5—C6—H6	120.3
C1—C2—C4	111.06 (18)	C8—C7—C6	120.1 (2)
C1—C2—C3	111.06 (18)	C8—C7—H7	119.9
C4—C2—C3	112.42 (18)	C6—C7—H7	119.9
C1—C2—Br1	106.84 (16)	C7—C8—C9	121.1 (2)
C4—C2—Br1	109.42 (14)	C7—C8—Cl1	119.44 (18)
C3—C2—Br1	105.74 (14)	C9—C8—Cl1	119.49 (18)
O1—C3—N1	124.1 (2)	C8—C9—C10	119.2 (2)
O1—C3—C2	120.33 (19)	C8—C9—H9	120.4
N1—C3—C2	115.56 (18)	C10—C9—H9	120.4
C2—C4—H4A	109.5	C9—C10—C5	120.3 (2)
C2—C4—H4B	109.5	C9—C10—H10	119.9
H4A—C4—H4B	109.5	C5—C10—H10	119.9
			
C5—N1—C3—O1	3.7 (3)	C10—C5—C6—C7	0.1 (3)
C5—N1—C3—C2	−179.28 (19)	N1—C5—C6—C7	−177.7 (2)
C1—C2—C3—O1	1.6 (3)	C5—C6—C7—C8	−0.4 (3)
C4—C2—C3—O1	126.7 (2)	C6—C7—C8—C9	0.3 (3)
Br1—C2—C3—O1	−113.94 (19)	C6—C7—C8—Cl1	−178.54 (17)
C1—C2—C3—N1	−175.6 (2)	C7—C8—C9—C10	0.1 (3)
C4—C2—C3—N1	−50.5 (2)	Cl1—C8—C9—C10	178.97 (17)
Br1—C2—C3—N1	68.9 (2)	C8—C9—C10—C5	−0.4 (3)
C3—N1—C5—C10	155.0 (2)	C6—C5—C10—C9	0.3 (3)
C3—N1—C5—C6	−27.1 (3)	N1—C5—C10—C9	178.2 (2)

### Hydrogen-bond geometry (Å, °)

**Table:**

*D*—H···*A*	*D*—H	H···*A*	*D*···*A*	*D*—H···*A*
N1—H1N···O1^i^	0.81 (3)	2.17 (3)	2.972 (2)	169 (3)
C10—H10···O1^i^	0.95	2.71	3.433 (3)	133.
C4—H4B···O1^i^	0.98	2.53	3.453 (3)	158.

Symmetry codes: (i) −*x*+3/2, *y*+1/2, *z*.
